# Effect of juvenile social isolation on excitability of prefrontal pyramidal cells with different subcortical axonal projections

**DOI:** 10.3389/fncel.2025.1549352

**Published:** 2025-05-30

**Authors:** Yosuke Nishihata, Hiroki Yoshino, Yoichi Ogawa, Taketoshi Sugimura, Kazuya Okamura, Sohei Kimoto, Kazuhiko Yamamuro, Manabu Makinodan, Yasuhiko Saito, Toshifumi Kishimoto

**Affiliations:** ^1^Department of Psychiatry, Nara Medical University, Kashihara, Japan; ^2^Mie Prefectural Mental Medical Center, Tsu, Japan; ^3^Department of Neurophysiology, Nara Medical University, Kashihara, Japan; ^4^Department of Neuropsychiatry, Wakayama Medical University, Wakayama, Japan; ^5^Center for Health Control, Nara Medical University School of Medicine, Kashihara, Japan; ^6^Department of Psychiatry, Fujita Health University School of Medicine, Toyoake, Japan

**Keywords:** social isolation, prefrontal cortex, pyramidal cells, thalamus, striatum, pontine nuclei

## Abstract

**Background:**

Social experience during development is crucial for the functional maturation of the prefrontal cortex (PFC). Juvenile social isolation (JSI) causes severe PFC dysfunction. JSI reduces intrinsic excitability and excitatory synaptic inputs for a subtype of layer-5 (L5) pyramidal cells showing prominent h-current (PH cells) in the medial PFC. PH cells do not have commissural or associational cortical output; instead, they project into subcortical areas. However, which subcortical area is the projection target of L5 pyramidal cells affected by JSI remains unascertained.

**Methods:**

Using retrograde neuronal tracing, we identified L5 pyramidal cells having three different projection targets: the mediodorsal thalamus, striatum, or pontine nuclei. We elucidated differences in functional properties among the three subclasses of L5 pyramidal cells and examined how JSI affects the intrinsic membrane properties and excitatory inputs for each class of L5 pyramidal cells.

**Results:**

Pyramidal cells projecting to the pontine nuclei had more excitatory synaptic inputs and more distinguishing intrinsic properties than pyramidal cells projecting to the mediodorsal thalamus and striatum. JSI increased the firing responsiveness of pyramidal cell projecting to mediodorsal thalamus and reduced excitatory synaptic inputs only onto pyramidal cells projecting to the pontine nuclei.

**Conclusion:**

JSI affects the development of L5 pyramidal cells based on their projection target.

## 1 Introduction

The prefrontal cortex (PFC) has undergone significant evolution in civilized animals, especially humans, wherein it plays a key role in cognitive functions, including decision-making, problem solving, abstract reasoning, social interaction, and planning (Miller and Cohen, 2001; Wood and Grafman, 2003). PFC dysfunction is associated with psychiatric disorders, such as schizophrenia, depression, attention-deficit hyperactivity disorder, and posttraumatic stress disorder (Shin et al., 2004; Brennan and Arnsten, 2008; Gonzalez-Burgos and Lewis, 2008; Pizzagalli and Roberts, 2022). Inadequate experience in infancy affects the ontogenetic development of the PFC and causes deficits in PFC-dependent functions in adulthood (Danese and McEwen, 2012). In Romanian orphans, imaging studies revealed that institutionalization decreased the functional (glucose metabolic rate) activity of the PFC and induced abnormal changes in a fiber tract connecting the PFC to the temporal lobe, which conferred deficits in cognitive and social abilities (Chugani et al., 2001; Eluvathingal et al., 2006).

In addition, in non-human mammals, juvenile social experiences affect brain development and subsequent behavioral characteristics (Freedman et al., 1961; Kolb et al., 2012; Bijlsma et al., 2023). Behavioral studies on rodents have shown that isolated rearing impairs cognitive functions later in adulthood (Han et al., 2011; Shao et al., 2013), disturbs social communication, and facilitates violent aggression in adulthood (Tóth et al., 2008). Juvenile social isolation (JSI) decreased approach behaviors and increased avoidance behaviors in adulthood (Hol et al., 1999). Insufficient juvenile social experiences have lasting effects on PFC structure and function that persist into adulthood. In rats, isolated rearing increases brain-derived neurotrophic factor protein levels (Han et al., 2011), decreases dopamine turnover (Heidbreder et al., 2000), and causes cortex volume loss (Day-Wilson et al., 2006) in the mPFC. Socially isolated mice exhibit myelination suppression within the mPFC as well as deficits in PFC-dependent behavioral abilities, which do not recover with reintroduction into a social environment (Makinodan et al., 2012). These findings suggest that JSI specifically affects post-weaning PFC development and induces abnormalities in biochemical and physiological aspects of PFC function and PFC-dependent behavioral abilities.

Layer 5 (L5) pyramidal cells constitute the primary source of cortical output (Gabbott et al., 2005; Morishima and Kawaguchi, 2006) and depending on their projection to target brain regions, have distinct interconnectivity, morphology, and firing patterns (Molnár and Cheung, 2006; Morishima and Kawaguchi, 2006; Otsuka and Kawaguchi, 2008; Brown and Hestrin, 2009). L5-mPFC pyramidal cells have been classified into two types (Dembrow et al., 2010; Gee et al., 2012): one subtype displays prominent hyperpolarization-activated cation currents (Ih), projects axons to subcortical areas, and has thicker apical dendrites and more primary branches, whereas the other subtype is characterized by a lack of prominent Ih, projects to the contralateral cortex, and has thinner apical dendrites and less primary branches. Lee et al. (2014) demonstrated that the two types of L5-pyramidal cells differ from each other in presynaptic excitatory and inhibitory inputs as well as in intrinsic membrane properties and projection areas.

By defining L5-mPFC pyramidal cells with prominent Ih as PH cells and those without prominent Ih as non-PH cells, we showed that JSI reduced excitatory synaptic inputs and the excitability of only PH cells, but not non-PH cells (Yamamuro et al., 2018). As the PH cell – a population of L5-mPFC pyramidal cell with prominent Ih – projects to various subcortical areas, PH cells can be further classified by their subcortical axonal projection area. Each subclass of PH cells might form distinct neural circuit by its distal projection and be involved in specific functions. Juvenile social experience may not universally promote the maturation of all PH cells, but specifically prompt some subclasses of PH cells that constitute the neural circuit for social and cognitive abilities. Lack of social experiences might disturb the development of the specific class of PH cells. However, it is unclear how L5-mPFC pyramidal cells functionally differentiate depending on their subcortical axonal projection area. The JSI-induced functional influences on each subclass of L5-mPFC pyramidal cells remain unelucidated. We focused on the mediodorsal thalamus, striatum, and pontine nuclei, which are candidate subcortical projection areas that are related to social function.

L5-mPFC pyramidal cells with axonal projections to the mediodorsal thalamus have a prominent Ih, which is similar to that in PH cells (Gee et al., 2012). The mediodorsal thalamus is not purely a relay nucleus that sends subcortical afferent inputs to the PFC, but rather a dominant projection target of the PFC (Goldman-Rakic and Porrino, 1985; Xiao et al., 2009). The neural loop circuit formed with this reciprocal connection is likely essential for cognitive function, including working memory (Parnaudeau et al., 2018). Furthermore, mPFC–mediodorsal thalamus connectivity plays a key role in social dominance (Wang et al., 2011; Zhou et al., 2017).

The striatum receives axonal projections from L5-mPFC pyramidal cells as well as other cortical areas (Anastasiades and Carter, 2021) and may be involved in the neural circuit for reward or reinforcement processing. In humans, functional magnetic resonance imaging (fMRI) studies have shown that striatal activity is influenced by diverse social events, including the evaluation of social rewards (Delgado, 2007; Izuma et al., 2008), cooperation with others (Rilling et al., 2002), social comparison with others (Fliessbach et al., 2007), and social reinforcement learning (Jones et al., 2011). Interestingly, pharmacological mPFC inactivation and the blockade of glutamatergic inputs to the striatum suppress and facilitate social play behavior, respectively, in adolescent rats (Van Kerkhof et al., 2013), which suggests that social interactions among young animals are modulated by PFC–striatum synergistic activity.

Besides its crucial role in motor control, the cerebellum mediates cognitive, social, and emotional abilities (Baumann et al., 2015; Hoche et al., 2016; Rudolph et al., 2023). Human studies have reported that patients with cerebellar damage show deficits in social–emotional cognition (Schmahmann, 2010). In primates, the PFC, similar to the primary motor cortex, connects reciprocally with the cerebellum through multi–synaptic relay, which forms a cerebellar–cortical closed loop (Kelly and Strick, 2003). The pontine nuclei relay cortical activity to the cerebellar cortex (Moya et al., 2014). Furthermore, the L5-mPFC pyramidal cell with axonal projections to the pontine nuclei has prominent Ih (Dembrow et al., 2010). Therefore, the L5-mPFC pyramidal cell–based activation of the mediodorsal thalamus, striatum, and pontine nuclei may be crucial for the execution of cognitive and social functions. Moreover, JSI-induced behavioral dysfunction is potentially attributable to the maldevelopment of L5-mPFC pyramidal cells that project to these areas.

We aimed to investigate the effect of JSI on the excitability of L5-mPFC pyramidal cells with axonal projections to the mediodorsal thalamus, striatum, and pontine nuclei for each. Therefore, using retrograde neural tracers and the whole-cell patch-clamp technique to measure the membrane properties and excitatory synaptic inputs for each pyramidal cell, we first identified L5-mPFC pyramidal cells that project to each of the three subcortical areas and characterized the electrophysiological properties for three subclasses of L5-mPFC pyramidal cells to examine how JSI affects the properties of three subclasses of L5-mPFC pyramidal cells.

## 2 Materials and methods

### 2.1 Animals and housing conditions

All experimental procedures were approved by the animal care and use committee of the Nara Medical University, and performed according to the institutional guidelines. Male C57/BL6 mice were used. All mice were maintained in a fixed 12-h light–dark cycle. After weaning on postnatal day 21 (P21), 4 male littermates were randomly assigned to 1 isolated and 3 group-reared subgroups. The isolated mouse was individually housed from P21 to P35, and thereafter, re-housed with its littermate. The non-isolation period lasted until P60–P70.

### 2.2 Bead infusion

To selectively label pyramidal cells that project to the three different subcortical areas, we injected retrogradely transported fluorescently labeled latex microspheres (Red or Green Retrobeads, Lumafluor, Naples) to each projection area. At P60–P70, mice were anesthetized with 2% isoflurane and mounted on a stereotactic apparatus (SR-6 M-HT, Narishige, Japan). Then, we exposed the skull surface and drilled holes of approximately 1-mm diameter over the injection site, which were situated according to stereotaxic coordinates as follows: mediodorsal thalamus: −1.5 mm anteroposterior relative to bregma (AP), +0.3 mm mediolateral (ML) and −3.5 mm dorsoventral (DV); striatum: +1.2 mm AP, −1.5 mm ML, and −3.8 mm DV; and pontine nuclei: −4.2 mm AP, −0.5 mm ML, and −5.5 mm DV. The retrograde tracer (300 nL) was injected using a glass capillary with a tip diameter of 30–40 μm at a rate of 100 nL/min. The injection glass capillary was left in place for 5 min to prevent backflow and then slowly removed. For each animal, either of two different-colored tracers were injected into either of the three different sites at each side of the brain. Choices for injection site and side were regulated to minimize any unevenness of sample size. Animals were allowed to recover from intracranial injection for a minimum of 3 (range 3–7) days before undergoing electrophysiological experiments. At the time of brain-slice preparation for electrophysiological experiments, we visually verified that retrograde tracer injections were targeted appropriately in each subcortical area. Electrophysiological recordings were obtained for pyramidal cells having ipsilateral projection.

### 2.3 Brain-slice preparation and electrophysiological recordings

Brain slices, including the mediofrontal cortex (prelimbic and infralimbic regions), were prepared using 60- to 70-day-old mice. The mouse was deeply anesthetized with isoflurane and decapitated. The brain was quickly removed and immersed in ice-cold sucrose-based solution bubbled with a mixed gas of 95% O_2_/5% CO_2_, containing (in mM): sucrose 230, KCl 2.5, NaHCO_3_ 25, NaH_2_PO_4_ 1.25, CaCl_2_ 0.5, MgSO_4_ 10, and D-glucose 10. Using a vibrating tissue slicer (Vibratome 1,000 Plus 102, Pelco International), the frontal cerebrum was sectioned into 330 μm-thick slices in a pseudo-coronal plane, slightly oblique along the horizontal plane. Immediately after sectioning, the slices were incubated for at least 60 min in a beaker filled with a standard artificial cerebrospinal fluid (ACSF) continuously bubbled with a mixed gas, containing (in mM): NaCl 125, KCl 2.5, NaHCO_3_ 25, NaH_2_PO_4_ 1.25, CaCl_2_ 2.0, MgCl_2_ 1.0, and D-glucose 25 at 32°C, and then maintained in the ACSF at 25°C.

Following incubation, the slice was transferred to a recording chamber (volume, approximately 0.8 mL), and submerged with U-shaped platinum wire and nylon fibers. The slices were super-fused at a flow rate of 2 mL/min with the ACSF saturated with the mixed gas of 95% O_2_/5% CO_2_ at 32°C. Neurons in the slice were video-imaged with an upright microscope (DM6000FS, Leica) equipped with both epifluorescence illuminator and infrared differential interference contrast (IR-DIC) optics. The L5 pyramidal cells projecting each of three subcortical areas was first identified by its green or red fluorescence, and then its cell body was visualized using the IR-DIC optics for enabling pipette-tip manipulation. L5 pyramidal cells were voltage- or current-clamped in the conventional whole-cell configuration, using Multiclamp 700 A amplifier (Axon Instruments). Patch pipettes were pulled from borosilicate glass and filled with an intracellular solution containing (in mM) 141 K-gluconate, 4 KCl, 2 MgCl_2_, 2 Mg-ATP, 0.3 Na_2_-GTP, 0.2 EGTA, 10 HEPES, at pH 7.25 with KOH. In these ionic compositions for the ACSF and pipette solution, Cl^−^ equilibrium potential is calculated as −74 mV at 32°C, which is nearly equal to the reversal potential for GABA_A_ receptor-mediated current. The membrane potentials were corrected for 13 mV liquid junction potential measured according to the method of Neher (1992). Data acquisition and stimulation were controlled by Signal 4 software with Power 1,401 interface equipment (Cambridge Electronic Design).

### 2.4 Voltage-clamp recording

To examine excitatory inputs onto L5 pyramidal cells, we recorded excitatory postsynaptic currents (EPSCs) in voltage-clamp mode. The pipette capacitance was adjusted for, whereas series resistance was continuously monitored without adjustments made. Only recordings with a stable series resistance of < 20 MΩ were included in our analysis. The current signals were low-pass filtered at 600 Hz and digitized at a sampling frequency of 10 kHz. To observe sEPSCs in their natural conditions during both excitatory and inhibitory activity, we did not add a GABA_A_ receptor antagonist into the ACSF; instead we inhibited the GABA_A_ receptor-mediated current solely for the recorded pyramidal cell. We set the holding potential for the L5 pyramidal cell at −70 mV. Under the recording configuration mentioned above, the GABA_A_ receptor-mediated postsynaptic currents were directed outward and were generally too small to be detected, whereas EPSCs were detected as definite inward currents. We also recorded tetrodotoxin (TTX)-resistant miniature EPSCs (mEPSC) in the presence of 10-μM gabazine and 1-μM TTX.

### 2.5 Current-clamp recording

To examine the subthreshold and action potential membrane properties of L5 pyramidal cell, we recorded membrane potentials in the current-clamp mode from recordings, wherein the series resistance was monitored and canceled using a bridge circuit, and pipette capacitance was compensated. Voltage signals were low-pass filtered at 10 kHz and digitized at 20 kHz. With a current injection, the baseline membrane potential was maintained near −70 mV. To examine action potential and subthreshold membrane properties, we recorded membrane potential responses to hyperpolarizing and depolarizing current pulses (500-ms duration). Depolarizing current pulses with intensities of 10–200 pA were injected in increments of 10 pA. Hyperpolarizing currents pulses with intensities of −10 to −200 pA were injected at a −10-pA step. Current pulse injections were repeated three times at each intensity.

**Figure 1:**
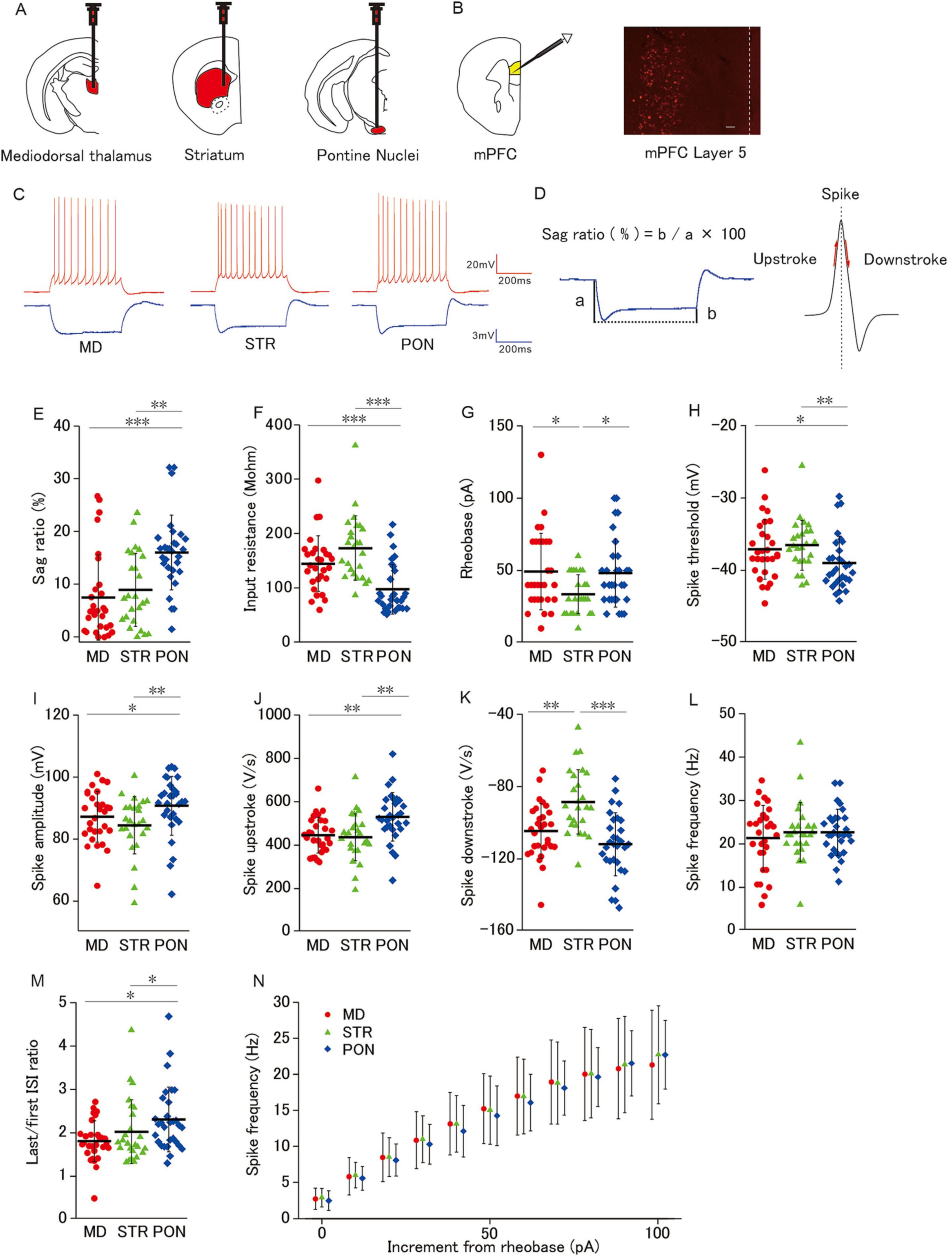
Membrane properties of mPFC L5 pyramidal cells classified by subcortical axonal projection areas (mediodorsal thalamus, striatum, and pontine nuclei). **(A)** Schemas showing neural tracer (Retrobeads) injection into three subcortical areas (Left: Mediodorsal thalamus, Middle: Striatum, and Right: Pontine nuclei). **(B)** Left: A schema showing whole-cell patch-clamp recordings from L5 pyramidal cells in mPFC. Right: a photomicrograph of Red Retrobeads labeled cells. Scale bars, 100 μm. Dot line, Pia. **(C)** Representative spikes elicited by a current injection of 100-pA larger than the rheobase (red) and −50 pA (blue) current injection from MD cells (left; L5 pyramidal cell-projecting axon to the mediodorsal thalamus), STR cells (middle; L5 pyramidal cell-projecting axon to the striatum), and PON cells (right; L5 pyramidal cell projecting axon to pontine nuclei). **(D)** Left: Representative trace shows a prominent voltage sag generated by a hyperpolarizing current injection (−50 pA, 500 ms). Sag ratio (%) = b/a × 100. Right: The maximum and the minimum of the spike slope were defined as the spike upstroke and downstroke, respectively. **(E)** The sag ratio of PON cells was significantly higher than that of MD cells (*p* < 0.001) and STR cells (*p* < 0.01). **(F)** The input resistance of PON cells was significantly lower than that of both MD and STR cells (both *p* < 0.001). **(G)** The rheobase of STR cells was significantly smaller than that of MD and PON cells (both *p* < 0.05). **(H)** PON cells had a significantly lower spike threshold than MD cells (*p* < 0.05) and STR cells (*p* < 0.01). **(I)** PON cells had a significantly larger spike amplitude than that of MD cells (*p* < 0.05) and STR cells (*p* < 0.01). **(J)** The spike upstroke of PON cells was significantly steeper than that of MD and STR cells (both *p* < 0.01). **(K)** The spike downstroke of STR cells was significantly more gradual than that of MD cells (*p* < 0.01) and PON cells (*p* < 0.001). **(L)** There was no significant difference in spike frequency among MD, STR and PON cells. **(M)** The last/first ISI ratio (the ratio between the last and first interspike interval) of PON cells was significantly higher than that of MD and STR cells (both *p* < 0.05). **(N)** There was no significant difference in the spike frequency–current curves across MD, STR, and PON cells. **(E–N)** Recorded cells and mice per group: MD cells (*n* = 28–29, 15 mice), STR cells (*n* = 25, 11 mice), and PON cells (*n* = 30, 14 mice). Data are presented as mean ± SD (dashed line and error bars) with individual data plots. *p < 0.05, **p < 0.01, ***p < 0.001. Full statistical results are provided in Supplementary Table 1.

We assessed the h-current magnitude to determine the voltage sag at hyperpolarization induced by a −50-pA current injection and calculated the sag ratio (sag ratio = b/a × 100; Figure 1D). The input resistance was estimated using the linear regression coefficient for peak-voltage changes induced by the injected hyperpolarizing currents (−50 to −10 pA, 500 ms). The rheobase (current threshold for excitation) was defined as the minimum current value at which the current injection elicited at least an action potential, and its action potential threshold was measured using the first derivative of the voltage curve. Thus, the action potential threshold was defined as the voltage at which the slope of potential trace just reached 10 mV/ms. Spike amplitude was defined as the voltage from the threshold to the peak of the action potential at the rheobase. The rheobase current and voltage threshold are indicative of excitability, that is, the ability to detect a small input. Furthermore, we measured the frequency of action potential in response to a depolarizing current injection at a 100 pA larger than the rheobase. We averaged the spike frequency obtained from three repetitions of current injection, and measured spike upstroke and downstroke, which were defined as the maximum and the minimum of the slope of action potential, respectively (Figure 1D). The spike upstroke is an index of sodium channel availability (Kodama et al., 1987).

We categorized pyramidal cells with >5% sag ratio as prominent h-current cells (PH cells), and the other cells as non-PH cells (Yamamuro et al., 2018).

### 2.6 Statistical analysis

We analyzed the membrane potential data obtained from the current-clamp recordings using Signal 4 software (Cambridge Electronic Design) and evaluated the subthreshold membrane and action potential properties of each cell. We used Mini Analysis software (Synaptosoft) to detect and analyze sEPSCs and mEPSCs on the membrane current data obtained from the voltage-clamp recordings. For each cell, all EPSCs for 5–20 min (at least 200 events were included in each recording duration) were detected and the mean amplitude and frequency were calculated. We used scatterplots for the data of intrinsic membrane properties to explicitly show the variations. The horizontal bar represents the mean. To derive the data for spike frequency, we used standard dot plots for each group, wherein each dot and error bar represent the group mean and standard deviation (SD; Figures 1N, 3K, 4K–M,V,W,X).

**Figure 2:**
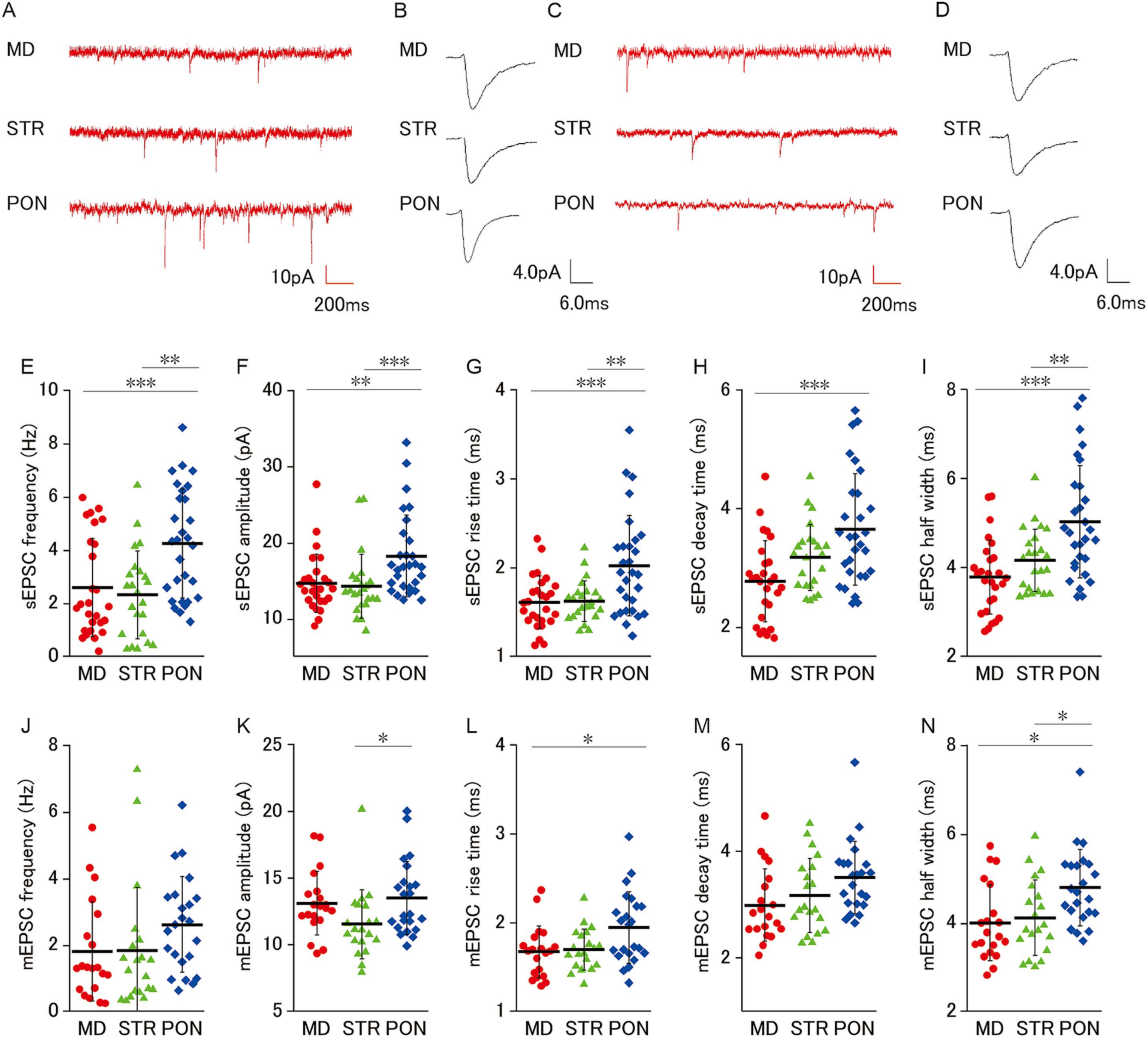
Characteristics of excitatory synaptic inputs on MD, STR, and PON cells. **(A)** Representative traces showing sEPSCs recorded from MD, STR, and PON cells. **(B)** Representative examples of sEPSCs in MD, STR, and PON cells. **(C)** Representative traces showing mEPSCs recorded from MD, STR, and PON cells. **(D)** Representative examples of mEPSCs in MD, STR, and PON cells. **(E)** The sEPSC frequency of PON cells was significantly higher than that of MD cells (*p* < 0.001) and STR cells (*p* < 0.01). **(F)** The sEPSC amplitude of PON cells was significantly larger than that of MD cells (*p* < 0.01) and STR cells (*p* < 0.001). **(G)** The sEPSC rise time of PON cells was significantly longer than that of MD cells (*p* < 0.001) and STR cells (*p* < 0.01). **(H)** The sEPSC decay time of PON cells was significantly longer than that of MD cells (*p* < 0.001), while the difference with STR cells was not significant. **(I)** The sEPSC half width of PON cells was significantly wider than that of MD cells (*p* < 0.001) and STR cells (*p* < 0.01). **(E–I)**, Recorded cells and mice per group: MD cells (*n* = 27, 15 mice); STR cells (*n* = 23, 11 mice); PON cells (*n* = 29, 14 mice). **(J)** There was no significant difference in the mEPSC frequency among MD, STR and PON cells. **(K)** The mEPSC amplitude of PON cells was significantly larger than that of STR cells (*p* < 0.05), while the difference with MD cells was not significant. **(L)** The mEPSC rise time of PON cells was significantly longer than that of MD cells (*p* < 0.05), while the difference with STR cells was not significant. **(M)** There was no significant difference in the mEPSC decay time among MD, STR and PON cells. **(N)** The mEPSC half width of PON cells was significantly wider than that of MD and STR cells (both *p* < 0.05). **(J–N)** Recorded cells and mice per group: MD cells (*n* = 20, 14 mice); STR cells (*n* = 20, 11 mice); PON cells (*n* = 23, 10 mice). Data are presented as mean ± SD (dashed line and error bars) with individual data plots. Error bars were adjusted to ensure that the lower limit does not extend below zero, as mEPSC frequency is a non-negative variable. *p < 0.05, **p < 0.01, ***p < 0.001. Full statistical results are provided in Supplementary Table 1.

**Figure 3:**
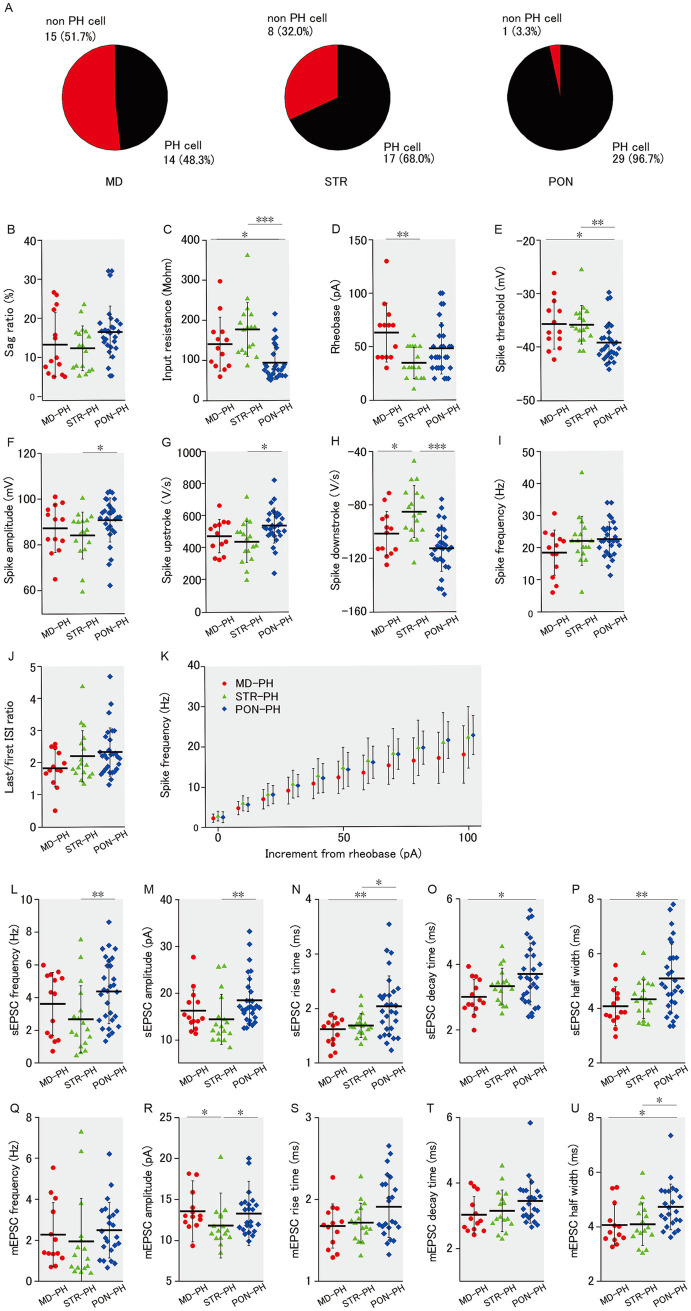
Membrane properties and excitatory synaptic inputs of PH cells within MD, STR, and PON cells. **(A)** Pie charts representing the proportion of PH cells in MD, STR, and PON cells. A significant association was observed among the three subclasses with respect to PH-cell prevalence (*p* < 0.001). **(B)** There was no significant difference in the sag ratio among three subclasses of PH cells. **(C)** The input resistance of PON-PH cells was significantly lower than that of MD-PH cells (*p* < 0.05) and STR-PH cells (*p* < 0.001). **(D)** The rheobase of STR-PH cells was significantly smaller than that of MD-PH cells (*p* < 0.01), while a comparison with PON-PH cells was not significant. **(E)** The spike threshold of PON-PH cells was significantly lower than that of MD-PH cells (*p* < 0.05) and STR-PH cells (*p* < 0.01). **(F)** The spike amplitude of PON-PH cells was significantly larger than that of STR-PH cells (*p* < 0.05), while a comparison with MD-PH cells was not significant. **(G)** The spike upstroke of PON-PH cells was significantly steeper than that of STR-PH cells (*p* < 0.05), while a comparison with MD-PH cells was not significant. **(H)** The spike downstroke of STR-PH cells was significantly more gradual than that of MD-PH cells (*p* < 0.05) and PON-PH cells (*p* < 0.001). **(I)** There was no significant difference in the spike frequency. **(J)** There was no significant difference in the last/first ISI ratio. **(K)** There was no significant difference in the spike frequency–current curves. **(B–K)** Recorded cells and mice per group: MD-PH cells (*n* = 13–14, 9 mice); STR-PH cells (*n* = 17, 10 mice); PON-PH cells (*n* = 29, 14 mice). **(L)** The sEPSC frequency of PON-PH cells was significantly higher than that of STR-PH cells (*p* < 0.01), while a comparison with MD-PH cells was not significant. **(M)** The sEPSC amplitude of PON-PH cells was significantly larger than that of STR-PH cells (*p* < 0.01), while a comparison with MD-PH cells was not significant. **(N)** The sEPSC rise time of PON-PH cells was significantly longer than that of MD-PH cells (*p* < 0.01) and STR-PH cells (*p* < 0.05). **(O)** The sEPSC decay time of PON-PH cells was significantly longer than that of MD-PH cells (*p* < 0.05), while a comparison with STR-PH cells was not significant. **(P)** The sEPSC half width of PON-PH cells was significantly wider than that of MD-PH cells (*p* < 0.01), with no significant difference found with STR-PH cells. **(L–P)** Recorded cells and mice per group: MD-PH cells (*n* = 14, 9 mice); STR-PH cells (*n* = 17, 10 mice); PON-PH cells (*n* = 29, 14 mice). **(Q)** There was no significant difference in the mEPSC frequency. **(R)** The mEPSC amplitude of STR-PH cells was significantly smaller than that of both MD-PH and PON-PH cells (both *p* < 0.05). **(S)** There was no significant difference in the mEPSC rise time. **(T)** There was no significant difference in the mEPSC decay time. **(U)** The mEPSC half width of PON-PH cells was significantly wider than that of MD-PH and STR-PH cells (both *p* < 0.05). **(Q–U)** Recorded cells and mice per group: MD-PH cells (*n* = 14, 9 mice); STR-PH cells (*n* = 16, 10 mice); PON-PH cells (*n* = 23, 10 mice). Data are presented as mean ± SD (dashed line and error bars) with individual data plots. Error bars were adjusted to ensure that the lower limit does not extend below zero, as mEPSC frequency is a non-negative variable. *p < 0.05, **p < 0.01, ***p < 0.001. Full statistical results are provided in Supplementary Table 1.

To determine the statistical intergroup difference, we used an open-source program, JASP,^1^ for statistical analyses. We initially applied the Shapiro–Wilk test to check the normality of the data distribution. In cases where the normality of the data distribution was rejected, the Shapiro–Wilk test was reapplied to the transformed data with natural logarithmic function. When the assumption of normality for the distribution of raw or log-transformed data was justified, group means were compared using Student’s *t* test, one-way ANOVA followed by Tukey’s honest significant difference (HSD) test. Conversely, when the normality of the distribution of the log-transformed data was rejected, we applied a nonparametric test (Mann–Whitney *U* test or Kruskal–Wallis test) followed by Dunn’s test. For repeated-measures data, multivariate analysis of variance (MANOVA) was applied when the variance/covariance matrix was not circular or when the assumption of equality between the variance/covariance matrices was rejected. For MANOVA, we used IBM SPSS Statistics version 26 (OS: Windows 10). For intergroup comparison of the occupancy proportion of PH cells, we used the chi-square test. To examine intergroup differences in the distribution of the sag ratio, we used the Kolmogorov–Smirnov test. Intergroup differences were considered significant if *p* < 0.05. The Supplementary Tables 1–3 list the *p*-value and the statistical test applied to each observed data type in this experiment.

## 3 Results

### 3.1 Three subcortical axonal projection-based L5 pyramidal cell subclasses with different action potentials and subthreshold membrane properties in group-reared animals

We first characterized the electrophysiological properties for three subclasses of L5 pyramidal cells classified by their subcortical projection area. In mPFC layer 5, populations of pyramidal cells projecting axons to different subcortical areas are intermingled with each other, and only a small minority of cells have multiple projection targets (Gabbott et al., 2005; Morishima and Kawaguchi, 2006). Retrograde tracing allows for the identification of each pyramidal cell projecting a particular subcortical region (Figures 1A,B). We examined the action potential and subthreshold membrane properties among L5 pyramidal cells with axonal projections to the mediodorsal thalamus (MD cells; *n* = 29), striatum (STR cells; *n* = 25), and pontine nuclei (PON cells; *n* = 30; Figure 1C). These cells were obtained from group-reared animals.

The sag ratio of PON cells was significantly higher than that of MD and STR cells (Figures 1D,E), which indicated that IH is more prominent in PON than in MD and STR cells. The input resistance of PON cells was significantly lower than that of MD and STR cells (Figure 1F). Regarding the spiking response to depolarizing current injection, PON cells showed distinctive characteristics from that of the other subclasses. The rheobase of STR cells was significantly smaller than that of MD and PON cells (Figure 1G). Compared to MD and STR, PON cells had significantly lower spike threshold (Figure 1H), significantly larger spike amplitude (Figure 1I), significantly steeper spike upstroke (Figures 1D,J). These results prove that PON cells have higher excitability than MD and STR. Evaluation of the spiking responsivity to the supra-threshold increment of depolarizing current showed no differences among the three subclasses neither in the curve of the relationship between spike frequency and current increment from rheobase, nor in spike frequency during the current injection of 100 pA above the rheobase (Figures 1L,N). Nonetheless, the last/first ISI ratio of PON cells was significantly higher than that of MD and STR cells (Figure 1M), which indicates that PON cells are more capable to sustain high-frequency firings than the other subclasses. Furthermore, the spike downstroke of STR was significantly gentler than that of MD and PON (Figures 1D,K), which might be related to the relatively smaller spike amplitude of STR pyramidal cells.

### 3.2 PON cells receive more excitatory synaptic inputs than MD and STR cells

We examined excitatory synaptic inputs from three subclasses of L5 pyramidal cells and found that PON cells apparently receive more excitatory inputs (Figure 2E). For sEPSC (Figures 2A,B), PON cells had significantly higher frequency and larger amplitude, longer rise time (10–90%) and longer half width than MD and STR (Figures 2E–G,I), and PON cells had significantly longer decay time (90–10%) than MD cells (Figure 2H). Furthermore, we analyzed TTX-resistant miniature EPSCs (mEPSCs), which represent the action potential-independent quantal transmitter release onto the recorded cell (Figures 2C,D). Although no significant intergroup differences in mEPSC frequency was observed among MD, STR, and PON cells (Figure 2J), the mEPSC amplitude of PON cells was significantly larger than that of STR cells (Figure 2K) and the mEPSC rise time of PON cells was significantly longer than that of MD cells (Figure 2L). There was no significant difference in the mEPSC decay time among MD, STR, and PON cells (Figure 2M). The mEPSC half width of PON cells was significantly wider than that of MD and STR cells (Figure 2N). These results regarding mEPSC suggest that the electrical charge per PSC for PON cells is larger than those in MD and STR cells, which suggests that the unitary synaptic event on PON cells has more potent action for excitation than those on the other subclasses of the pyramidal cell.

**Figure 4:**
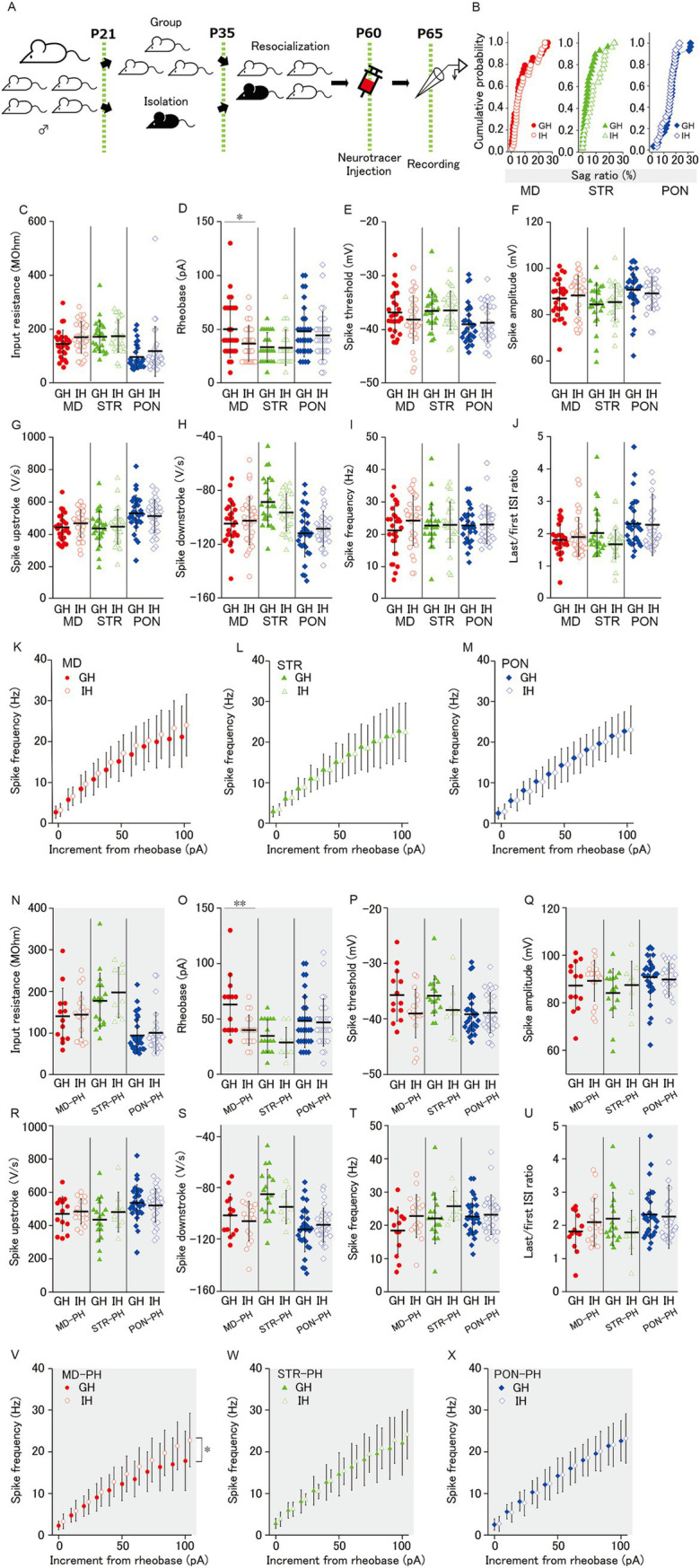
Effect of juvenile social isolation on membrane properties of MD/MD-PH, STR/STR-PH, and PON/PON-PH cells. **(A)** Experimental design for juvenile social isolation (JSI; IH: isolated-housing mice, black, GH: group-housing mice, white). The isolated-housing mouse is bred alone during P21–35. Before and after the isolation, the isolated mouse is bred together with its group-housing littermates. **(B)** There was no effect of JSI on sag ratio of MD, STR, and PON cells. **(C, E-J)** There was no significant difference of MD, STR, and PON cells, between GH and IH mice, in **(C)** the input resistance, **(E)** the spike threshold, **(F)** the spike amplitude, **(G)** the spike upstroke, **(H)** the spike downstroke, **(I)** the spike frequency and **(J)** the last/first ISI ratio. **(D)** Social isolation significantly reduced the rheobase of MD cells (*p* < 0.05), but not of STR or PON cells. **(K–M)** The spike frequency–current curves did not differ significantly between GH and IH mice in MD, STR, or PON cells. **(B–M)** Recorded cells and mice per group: MD cells (GH: *n* = 28**–**29, 15 mice; IH: *n* = 34, 17 mice); STR cells (GH: *n* = 25, 11 mice; IH: *n* = 28, 14 mice); PON cells (GH: *n* = 30, 14 mice; IH: *n* = 32, 12 mice). **(N)** The input resistance in PH cells showed no significant difference between housing conditions. **(O)** Social isolation significantly reduced the rheobase of MD-PH cells (*p* < 0.01), but had no effect on STR-PH or PON-PH cells. **(P)** Although the spike threshold differences between GH and IH mice approached significance in STR-PH cells (*p* = 0.051) and also in MD-PH cells (*p* = 0.052), no statistically significant differences were observed in any group. **(Q–U)** The spike amplitude, spike upstroke, spike downstroke, spike frequency, and last/first ISI ratio in PH cells showed no significant difference between GH and IH mice. **(V–X)** JSI significantly shifted the spike frequency–current curves upward only in MD-PH cells (*p* < 0.05), with no significant changes observed in STR-PH or PON-PH cells. **(N–X)** Recorded cells and mice per group: MD-PH cells (GH: *n* = 13, 9 mice; IH: *n* = 18, 11 mice); STR-PH cells (GH: *n* = 17, 10 mice; IH: *n* = 9, 8 mice); PON-PH cells (GH: *n* = 29, 14 mice; IH: *n* = 29, 12 mice). Data are presented as mean ± SD (dashed line and error bars) with individual data plots. *p < 0.05, **p < 0.01, ***p < 0.001. Full statistical results are provided in Supplementary Table 2.

### 3.3 Functional differences in membrane properties and excitatory inputs were not attributable to heterogeneity in PH cell occupancy

In our previous study (Yamamuro et al., 2018), PH cells, that is mPFC L5 pyramidal cells with prominent h-current (>5% sag ratio), differed from non-PH cells in intrinsic membrane and action potential properties. Compared to non-PH cells, PH cells had lower input resistance, lower spike threshold, and larger spike amplitude as well as higher sEPSCs and mEPSC frequencies. Although mPFC L5 pyramidal cells with prominent h-current have axonal projections to subcortical areas (Dembrow et al., 2010; Gee et al., 2012), in this study, not all of the mPFC L5 pyramidal cells that had axonal projections to subcortical area showed prominent h-current, as observed from the voltage sag during hyperpolarizing current injection. Among the three subclasses of pyramidal cells classified by subcortical projection area, there was a significant difference in the occupancy of PH cells (Figure 3A). The occupancies of PH cells labeled with retrograde tracers were 48.3% (14/29) in MD, 68.0% (17/25) in STR, and 96.7% (29/30) in PON cells, respectively. Therefore, the differences in the occupancy of PH cell might confer differences among the three subclasses of pyramidal cells in membrane properties and excitatory synaptic input (Figures 1, 2, respectively). We reexamined the membrane properties and excitatory input only for PH cells that belonged to MD, STR, and PON subclasses (MD-PH, STR-PH and PON-PH cells); as expected, we found no differences in the sag ratio (Figure 3B) among the three subclasses of PH cells. The differences revealed in the comprehensive analyses, including non-PH cells, were mostly evident even when analyses were limited to PH cells. The input resistance of PON-PH cells was significantly lower than that of MD-PH and STR-PH cells (Figure 3C). The rheobase of STR-PH cells was significantly smaller than that of MD-PH cells (Figure 3D). The spike threshold of PON-PH cells was significantly lower than that of MD-PH and STR-PH cells (Figure 3E). The spike amplitude in PON-PH cells was significantly larger than that of STR-PH cells (Figure 3F). The spike upstroke in PON-PH cells was significantly steeper than that of STR-PH cells (Figure 3G), the spike downstroke of STR-PH cells was significantly gentler than that of MD-PH and PON-PH cells (Figure 3H). There was no intergroup difference among the three PH cell subclasses in the spiking responsivity to the supra-threshold increment of depolarizing current (Figures 3I,K) or the last/first ISI ratio (Figure 3J).

Regarding excitatory synaptic inputs onto PH cells, the results were similar to those of membrane properties. The sEPSC frequency for PON-PH cells was significantly higher than that for STR-PH cells (Figure 3L), the sEPSC amplitude for PON-PH cells was significantly larger than that for STR-PH cells (Figure 3M), the sEPSC rise time for PON-PH cells was significantly longer than that for MD-PH and STR-PH cells (Figure 3N), the sEPSC decay time for PON-PH cells was significantly longer than that for MD-PH cells (Figure 3O), and the sEPSC half width for PON-PH cells was significantly wider than that for MD-PH cells (Figure 3P). The mEPSC amplitude for MD-PH and PON-PH cells was significantly larger than that for STR-PH cells (Figure 3R), the mEPSC half width in PON-PH cells was significantly wider than that of MD-PH and STR-PH cells (Figure 3U). Among the three subclasses of PH cells, there were no significant intergroup differences in mEPSC frequency, rise and decay times (Figures 3Q,S,T).

A small number of the significant functional differences observed among MD, STR, and PON cells (Figures 1, 2) lacked significance when the analyses were restricted only to PH cells, which may be partly attributable to the lower power of the statistical test owing to the smaller sample size. Rather, these results indicated that the differences in membrane properties and excitatory inputs among the three subclasses of pyramidal cells are not solely attributable to the difference in the occupancy of PH cells, but rather that each of the three subclasses with different projection areas has its own electrophysiological features.

### 3.4 JSI affected action potential properties of MD, but not STR and PON, cells

JSI reduces the firing reactivity and excitatory synaptic input for mPFC L5 PH cells in adult mice (Yamamuro et al., 2018). As MD, STR, and PON cells included PH cells, JSI could affect the membrane properties and excitatory synaptic inputs of MD, STR, and PON cells. To confirm this possibility, we examined the differences in membrane properties between group-housing (GH) and isolated-housing (IH) mice (Figure 4A) for each of the three subclasses of pyramidal cells. However, there was no difference in the distribution of sag ratio and input resistance between GH and IH mice (Figures 4B,C) for MD, STR, and PON cells. Regarding the spiking response to depolarizing current injection, we found no significant differences between GH and IH for MD, STR, and PON cells in all the measures, except rheobase (Figure 4C,E–M) wherein the rheobase of MD cells in IH was significantly smaller than that of MD cells in GH mice, but not in STR and PON cells (Figure 4D).

As shown in Figure 3A, the occupancies of PH cells for MD and STR cells were only a half and two-thirds, respectively. We previously (Yamamuro et al., 2018) reported the effects of JSI on the membrane properties for PH cells but not for non-PH cells. Therefore, herein, we analyzed the membrane properties by focusing only on PH cells. Accordingly, for MD-PH cells, there were significant differences in rheobase and the spike frequency–current curve of GH and IH mice (Figures 4O,V). JSI significantly reduced the rheobase and altered the spike frequency–current curve to a steeper one for MD-PH cells. These two results indicate that MD-PH cells in IH mice can convert smaller current input into excitation and, thereafter, code smaller input change into an increase in firing rate compared to that in GH mice. In addition, the spike threshold of MD-PH and STR-PH cells for IH mice tended to be lower than those for GH mice, although the differences are only marginally and not entirely significant (Figure 4P). For the other measures on subthreshold membrane properties, there were no significant differences between GH and IH mice for MD-PH, STR-PH, and PON-PH cells (Figures 4N,P–U) or in rheobase and the spike frequency–current curve between GH and IH in STR-PH and PON-PH cells (Figures 4O,W,X).

These results indicate that JSI has no detectable effect on the intrinsic membrane properties for STR, STR-PH, PON and PON-PH cells whereas JSI increases the action potential reactivity of MD and MD-PH cells.

### 3.5 JSI decreases excitatory synaptic inputs to PON cells

**Figure 5:**
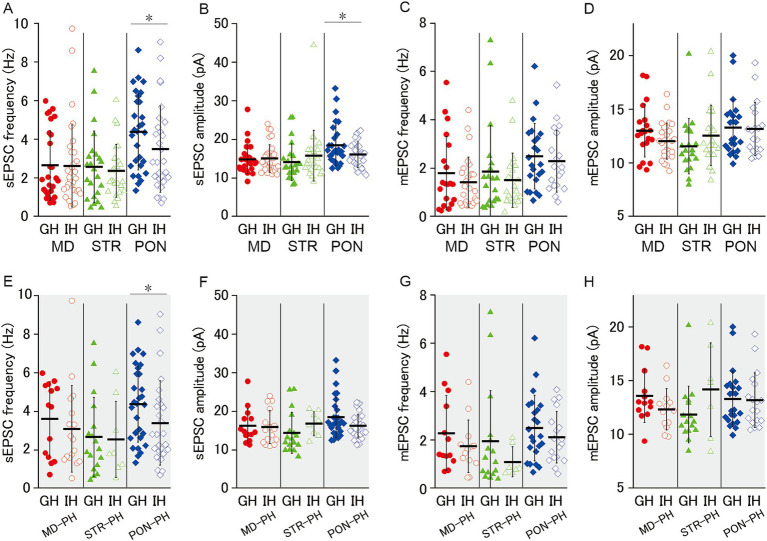
Effect of juvenile social isolation on excitatory synaptic inputs of MD/MD-PH, STR/STR-PH, and PON/PON-PH cells. **(A,B)** JSI significantly reduced both the sEPSC frequency and amplitude of PON cells (both *p* < 0.05), whereas there were no significant differences in MD or STR cells. Recorded cells and mice per group: MD cells (GH: *n* = 27, 15 mice; IH: *n* = 33, 17 mice); STR cells (GH: *n* = 23, 11 mice; IH: *n* = 26, 13 mice); PON cells (GH: *n* = 29, 14 mice; IH: *n* = 29, 12 mice). **(C,D)** There were no significant differences in either the mEPSC frequency or amplitude between GH and IH mice in MD, STR, or PON cells. Recorded cells and mice per group: MD cells (GH: *n* = 20, 14 mice; IH: *n* = 27, 15 mice); STR cells (GH: *n* = 20, 11 mice; IH: *n* = 22, 13 mice); PON cells (GH: *n* = 23, 10 mice; IH: *n* = 19, 9 mice). **(E–H)** JSI significantly reduced the sEPSC frequency in PON-PH cells (*p* < 0.05), whereas there were no significant differences in the sEPSC amplitude, mEPSC frequency, or mEPSC amplitude between GH and IH mice in PH cells. Recorded cells and mice per group: MD-PH cells (GH: *n* = 13, 9 mice; IH: *n* = 13, 8 mice); STR-PH cells (GH: *n* = 16, 10 mice; IH: *n* = 7, 6 mice); PON-PH cells (GH: *n* = 23, 10 mice; IH: *n* = 18, 9 mice). Data are presented as mean ± SD (dashed line and error bars) with individual data plots. Error bars were adjusted to ensure that the lower limit does not extend below zero, as mEPSC frequency is a non-negative variable. *p < 0.05, **p < 0.01, ***p < 0.001. Full statistical results are provided in Supplementary Table 2.

Our previous study revealed that JSI decreases EPSC excitatory synaptic input onto L5-PH cells in adult mice (Yamamuro et al., 2018). JSI could reduce excitatory synaptic inputs onto MD, STR, and PON cells. To investigate this possibility, we examined the effects of JSI for EPSCs onto MD, STR, and PON cells. In MD and STR cells, there was no significant differences in sEPSC frequency, sEPSC amplitude, mEPSC frequency, nor mEPSC amplitude between GH and IH mice (Figures 5A–F). In PON cells b, JSI significantly reduced sEPSC frequency and sEPSC amplitude, whereas JSI had no effect on mEPSC (Figures 5A–F). Even when the analyses were restricted to PH cells, JSI significantly reduced the sEPSC frequency of PON-PH cells, and its reducing effect on sEPSC amplitude was marginally significant (Figures 5E,F). GH and IH mice did not significantly differ regarding the frequency and amplitude of sEPSC or mEPSC in MD-PH and STR-PH cells (Figures 5E–H). These results indicate that JSI predominantly decreases action potential-dependent excitatory synaptic input to PON, but not MD and STR cells.

## 4 Discussion

In this study, we first characterized three different subclasses of L5-mPFC pyramidal cells with axonal projections to the mediodorsal thalamus, striatum, and pontine nuclei by examining their intrinsic membrane properties and excitatory synaptic input. We found a considerable variation in the magnitude of h-current among L5-mPFC pyramidal cells that project to subcortical areas. Whereas almost all the PON cells were PH cells, one third of STR and half of MD cells were not determined as PH cells based on the sag ratio (5% at a current injection of −50 pA). Furthermore, the three subclasses of pyramidal cells differed from each other in input resistance.

PON cells had distinct features from the other two classes, MD and STR cells. PON cells are characterized not only by their prominent h-current, low input resistance but also by high excitability. Generally, a depolarizing current injection to the cell with lower input resistance causes smaller depolarization, and a larger current is needed for the elicitation of action potential. In PON cells, this situation may be compensated by elevating the intrinsic excitability. PON cells had lower spike thresholds and showed action potentials with larger amplitudes and steeper rises (larger upstrokes) than those of the other two subclasses. This may represent the dense expression of voltage-dependent sodium channel on PON cells. Furthermore, PON cells had distinguishable features on excitatory input. Our analysis of the averaged waveform of both TTX-resistant and spontaneous EPSCs revealed that the electrical charge induced by a quantal transmitter release for PON cells is larger than MD and STR cells. This suggests that glutamatergic synapses with high unitary efficiency are formed on PON cells. Although the frequency of sEPSCs for PON cells was higher than those for MD and STR cells, no difference was shown in that of mEPSCs among the three cell subclasses, which indicates that only the excitation-dependent activations of synapses was more frequent in PON cells. This suggests that functional connections from the neighboring excitatory cells are dominantly formed onto PON cells. As sources of excitatory inputs to PON cells, inputs from layer 2/3 pyramidal neurons (Otsuka and Kawaguchi, 2008) and from pyramidal neurons projecting to the striatum (Morishima and Kawaguchi, 2006) can be considered. Furthermore, assuming that neurons projecting to the superior colliculus share similar properties with PON cells, excitatory inputs from neurons projecting to the contralateral cortex may also be possible (Brown and Hestrin, 2009). The abovementioned features of PON cell are well congruent with those of PH cells.

Since PH cells were much more prevalent among PON cells than among MD and STR cells, the observed differences in intrinsic membrane properties and excitatory inputs were initially thought to reflect this occupancy. However, further analysis limited to PH cells refuted this hypothesis. Most differences persisted even when comparing only PH cells across regions, indicating that intrinsic properties and synaptic inputs of L5 pyramidal neurons vary depending on their projection targets.

As previously reported (Dembrow et al., 2010; Gee et al., 2012), we found that mPFC L5 pyramidal cells with axonal projections to the pontine nuclei and mediodorsal thalamus showed large h-current. Furthermore, we reported that L5-mPFC pyramidal cells with striatal axonal projections showed large h-current values. STR cells, similar to MD cells, had a relatively high input resistance and low current threshold for excitation (rheobase). The excitatory input onto STR cells were low in both frequency and unitary size as compared with PON cells. These features were also evident for STR-PH cells. Unfortunately, at present we are unable to propose a hypothesis regarding the functional significance of these differentiated features of STR cells. However, these features may be related to the fact that STR cells, like MD cells, have relatively short-range of projections as compared with PON cells.

MD cells did not differ considerably from STR cells in their intrinsic membrane properties and excitatory input. However, further detailed analysis suggested that the MD cell population is functionally heterogeneous. Specifically, MD-PH cells exhibited significantly higher sEPSC amplitude and frequency compared to MD-non-PH cells, whereas there were no such differences between STR-PH and STR-non-PH cells (Supplementary Table 3). This indicates that the MD cell group may be inhomogeneous and comprise at least two functionally differentiated subgroups. This heterogeneity may obscure the differences in spike amplitude, ISI, and sEPSC frequency and amplitude between the PON-PH and MD-PH cells, in contrast to the significant differences observed between PON and MD (Figures 3F,G,J,L,M). It has been demonstrated that pyramidal neurons in the mPFC projecting to the mediodorsal thalamus can be divided into two types, each functionally contributing differently to cognitive behavioral control depending on whether they project to the lateral or medial MD (de Kloet et al., 2021). This heterogeneity seems important for resolving the mechanisms of JSI induced behavioral disorder, as discussed below. In addition, it is noteworthy that hyperpolarization-activated cyclic-nucleotide-gated channel (HCN), which mediates h-current, is related to the synaptic response of MD cells as well as to their intrinsic membrane properties. Anastasiades et al. (2018) have demonstrated that the pharmacological inhibition of HCN augments the relative amplitude EPSP and IPSP on MD cells in comparison to those on neighboring pyramidal cells projecting contralateral mPFC, which indicates that HCN may serve to normalize the magnitudes of excitatory and inhibitory synaptic voltage responses within this area. HCN expression level may be related to not only the intrinsic excitability but also the regulation of synaptic inputs of MD cells. The functional significance of variation in HCN expression within MD cell population, as well as within STR cells, should be examined in future studies.

The principal objective of our study was focused on the effects of JSI on intrinsic membrane properties and excitatory input for the three subclasses of L5 pyramidal cells having subcortical projection. Although for STR cells neither intrinsic membrane properties nor excitatory inputs was affected by JSI, we found that the effects of JSI on intrinsic membrane properties and excitatory inputs for MD cells differed from those for PON cells.

JSI decreased rheobase (current threshold for excitation) for both MD and MD-PH cells, and steeply inclined the curve of the relationship of elicited firing rate to injected current for MD-PH cells. These findings indicate that JSI elevates the excitability of MD and MD-PH cells in adulthood. This effect is not consistent with that observed in a previous study for whole PH cells (Yamamuro et al., 2018). In addition, this present finding contradicts another report that JSI reduces the excitability of mPFC cells projecting to the posterior paraventricular thalamus (pPVT) in adulthood (Yamamuro et al., 2020). Therefore, it is obvious that JSI induces different effects on the intrinsic membrane properties of mPFC pyramidal cells depending on their projection area even within thalamus. The elevation in the excitability of MD cells caused by JSI could exhibit some compensatory effect for the lowered excitability of the majority of mPFC pyramidal cells with prominent h-current. Interestingly, it has recently reported that excessive excitatory synaptic inputs onto mediodorsal thalamus disturb prepulse inhibition (PPI) (Kim et al., 2021). Impairment of PPI is the most typical behavioral alteration caused by social isolation (Fone and Porkess, 2008). Our previous study employing the same mouse-rearing protocol confirmed that JSI impairs PPI (Yamamuro et al., 2018). It is probable that the social isolation-induced elevation in the excitability of MD cells leads to an excessive excitatory input onto mediodorsal thalamic neurons and disturbs PPI. Recent studies using optogenetic techniques have demonstrated that the mPFC-to-mediodorsal thalamus projection is involved in behavioral control for various cognitive tasks requiring behavioral inhibition for a certain time or shift in action selection. The optogenetic time-limited inhibition of mPFC-to-mediodorsal thalamus synaptic transmission during the choice period, but not the delay period of delayed non-matching to sample T-maze task disturbs correct performance, which suggests that this projection is not primarily responsible for the maintenance of working memory, but rather plays a crucial role in contextual action selection (Bolkan et al., 2017). The activity of mPFC pyramidal cells projecting to mediodorsal thalamus, as well as those projecting to striatum, is crucial for switching behavior based on the outcome on a probabilistic reversal task (Nakayama et al., 2018). And the firings of PFC neurons projecting mediodorsal thalamus encode the outcome of behavioral selection, and serve cognitive flexibility on a cross-modal set-shifting task (Spellman et al., 2021). Furthermore, de Kloet et al. (2021) have demonstrated that the activity of the dorsomedial PFC neurons projecting to the lateral subregion of mediodorsal thalamus facilitate behavioral initiation (“Go”), whereas those projecting to the medial subregion of mediodorsal thalamus mediate behavioral inhibition (“Stop”), which suggests that two subpopulations of PFC neurons projecting to mediodorsal thalamus are involved in the tactical selection of behavior through the push-pull operation of the two. JSI induced an increase in the excitability of MD neurons, observed as reduced rheobase in our study, may excessively facilitate behavioral initiation and lead to inadequate action initiation or impulsive behavior, which is characteristic of attention-deficit hyperactivity disorder. Conversely, if hyperexcitability occurs in the neuron population involved in behavioral inhibition, it may underlie repetitive compulsive behaviors such as checking, which frequently observed in depression and obsessive-compulsive disorder.

As PON cells were representative PH cells, we expected that JSI should induce an evident decrease in the excitability of PON cells. Nevertheless, in this study, there was no detectable effect of JSI on the excitability of PON cells. However, JSI decreased spontaneous excitation-dependent synaptic events, which suggests the prohibition against the formation of functional excitatory synapses onto PON cells during post-weaning developmental period. Pontine nuclei serve as the principal relay center, receiving neocortical efferent activities and sending their information to cerebellar cortex. The cerebellum, in turn, communicates the processed output to the neocortex via cerebellar nuclei. This mutual connection between the neocortex and cerebellum has been considered to be involved mainly in motor control, especially in correcting errors. Recent studies have demonstrated the crucial role of the cerebellum in cognitive, social, and emotional abilities (Baumann et al., 2015; Hoche et al., 2016; Rudolph et al., 2023). Notably, human fMRI studies have shown that abnormalities in functional connections between the cerebellum and PFC are related to neurodevelopmental disorders (Fatemi et al., 2012), such as autism spectrum disorder (Ramos et al., 2018) and attention-deficit hyperactivity disorder (Mizuno et al., 2017). The normal functioning of the mPFC–cerebellum loop may be affected by social isolation-induced reduction in excitatory input onto PON cells, that is, the mPFC pyramidal cell connection to the cerebellar cortex via pontine nuclei. Such an alteration may underlie behavioral outcomes that arise from social isolation in infancy. Wu et al. (2018) have demonstrated that the optogenetic inhibition of caudal mPFC to pontine nuclei projection disturbs the acquisition of conditioned eyeblink response to a weak conditioned stimulus but not that to a strong conditioned stimulus. In this context, reduced excitatory input to PON cells induced by JSI, as shown in our study, may cause a failure in the functional transmission of mPFC–to-pontine nuclei. Such a failure could impair needful behavioral modulation guided by weak cues, which potentially results in the rigid and hypersensitive behavioral patterns observed in neurodevelopmental disorders such as autism spectrum disorder. This possibility that a failure in mPFC-to-pontine nuclei connection impairs behavioral modulation responding to weak stimuli may also underlie PPI deficit induced by JSI, since the functional ignorance of pre-pulse weak signal should disturb PPI deficit.

In this study, we found that social isolation during post-weaning period for 2 weeks differentially affected membrane properties of L5 pyramidal cells and excitatory inputs onto them depending on their projection area. However, JSI did not reduce intrinsic excitability for any subclass of mPFC pyramidal cells having subcortical projections, which is inconsistent with our previous study (Yamamuro et al., 2018). Therefore, future research should explore the possibility that another subgroup of L5 pyramidal cells—such as those projecting to the posterior paraventricular thalamus (pPVT), as demonstrated by Yamamuro et al. (2020)—exhibits reduced intrinsic excitability following JSI.

### 4.1 Conclusion

The electrophysiological properties of mPFC L5 pyramidal cells with axonal projections to the pontine nuclei are distinct from those of other L5 pyramidal cells projecting to the mediodorsal thalamus and striatum. JSI increases the action potential responsiveness of L5 pyramidal cells that project to the mediodorsal thalamus and reduces excitatory synaptic input onto L5 pyramidal cells that project to the pontine nuclei.

## Data Availability

The original contributions presented in the study are included in the article/Supplementary material, further inquiries can be directed to the corresponding author.

## Footnotes

^1^ https://jasp-stats.org/
