# Supplementary material for: Effect of juvenile social isolation on excitability of prefrontal pyramidal cells with different subcortical axonal projections
Source: Front Cell Neurosci. 2025 May 30;19:1549352. doi: 10.3389/fncel.2025.1549352 (PMC12163027; doi:10.3389/fncel.2025.1549352)
Supplement: Supplementary file 1 [file Data_Sheet_1.PDF]

Supplementary table 1. Comparison of electrophysiological properties among three subclasses of L5-mPFC pyramidal cells in group-housing mice

Variable	Figure	Shapiro-Wilk test	Shapiro-Wilk test after NLT*	Statistics		Post Hoc statistics			
				Statistical methods	p value	Statistical methods	p value		
							MD versus PON	MD versus STR	STR versus PON
Sag ratio (%)	1E	< .001	< .001	Kruskal-Wallis test	< .001	Dunn’s test	< .001	0.357	0.002
Input resistance (Mohm)	1F	< .001	0.500	One-way ANOVA※	< .001	Tukey’s HSD test	< .001	0.177	< .001
Rheobase (pA)	1G	< .001	0.071	One-way ANOVA※	0.016	Tukey’s HSD test	0.990	0.027	0.034
Spike threshold (mV)	1H	0.003	< .001	Kruskal-Wallis test	0.010	Dunn’s test	0.020	0.600	0.005
Spike amplitude (mV)	1I	< .001	< .001	Kruskal-Wallis test	0.011	Dunn’s test	0.033	0.433	0.004
Spike upstroke (V/s)	1J	0.278		One-way ANOVA	< .001	Tukey’s HSD test	0.005	0.974	0.003
Spike downstroke (V/s)	1K	0.824		One-way ANOVA	< .001	Tukey’s HSD test	0.236	0.003	< .001
Spike frequency (Hz)	1L	0.353		One-way ANOVA	0.596				
Last/first ISI ratio	1M	< .001	< .001	Kruskal-Wallis test	0.031	Dunn’s test	0.016	0.785	0.039
Spike frequency-current curves	1N			MANOVA	0.514				
sEPSC frequency (Hz)	2E	< .001	0.002	Kruskal-Wallis test	< .001	Dunn’s test	< .001	0.984	0.001
sEPSC amplitude (pA)	2F	0.002	0.374	One-way ANOVA※	< .001	Tukey’s HSD test	0.003	0.360	< .001
sEPSC rise time (ms)	2G	0.002	0.706	One-way ANOVA※	< .001	Tukey’s HSD test	< .001	0.949	0.002
sEPSC decay time (ms)	2H	0.026	0.258	One-way ANOVA※	< .001	Tukey’ s HSD test	< .001	0.057	0.086
sEPSC half width (ms)	2I	0.036	0.297	One-way ANOVA※	< .001	Tukey’s HSD test	< .001	0.191	0.007
mEPSC frequency (Hz)	2J	< .001	0.150	One-way ANOVA※	0.042	Tukey’s HSD test	0.071	0.997	0.085
mEPSC amplitude (pA)	2K	< .001	0.633	One-way ANOVA※	0.038	Tukey’s HSD test	0.922	0.115	0.042
mEPSC rise time (ms)	2L	0.045	0.322	One-way ANOVA※	0.034	Tukey’s HSD test	0.046	0.945	0.097
mEPSC decay time (ms)	2M	< .001	0.162	One-way ANOVA※	0.061				
mEPSC half width (ms)	2N	0.006	0.190	One-way ANOVA※	0.010	Tukey’s HSD test	0.014	0.897	0.046
Sag ratio (%)	3B	0.007	0.007	Kruskal-Wallis test	0.089				
Input resistance (Mohm)	3C	0.001	0.232	One-way ANOVA※	< .001	Tukey’s HSD test	0.015	0.174	< .001
Rheobase (pA)	3D	0.002	0.270	One-way ANOVA※	0.003	Tukey’s HSD test	0.146	0.002	0.084
Spike threshold (mV)	3E	0.001	< .001	Kruskal-Wallis test	0.003	Dunn’s test	0.025	0.789	0.007
Spike amplitude (mV)	3F	0.001	< .001	Kruskal-Wallis test	0.035	Dunn’s test	0.238	0.287	0.010
Spike upstroke (V/s)	3G	0.624		One-way ANOVA	0.014	Tukey’s HSD test	0.197	0.676	0.013
Spike downstroke (V/s)	3H	0.797		One-way ANOVA	< .001	Tukey’s HSD test	0.172	0.041	< .001
Spike frequency (Hz)	3I	0.432	0.003	Kruskal-Wallis test	0.142				
Last/first ISI ratio	3J	< .001	0.003	Kruskal-Wallis test	0.209				
Spike frequency-current curves	3K			MANOVA	0.191				
sEPSC frequency (Hz)	3L	0.026	0.239	One-way ANOVA※	0.006	Tukey’s HSD test	0.423	0.214	0.004
sEPSC amplitude (pA)	3M	< .001	0.002	Kruskal-Wallis test	0.008	Dunn’s test	0.183	0.159	0.002
sEPSC rise time (ms)	3N	0.004	0.593	One-way ANOVA※	0.005	Tukey’s HSD test	0.009	0.783	0.042
sEPSC decay time (ms)	3O	0.135		One-way ANOVA	0.021	Tukey’s HSD test	0.019	0.482	0.253
sEPSC half width (ms)	3P	0.095		One-way ANOVA	0.006	Tukey’s HSD test	0.009	0.677	0.067
mEPSC frequency (Hz)	3Q	< .001	0.388	One-way ANOVA※	0.084				
mEPSC amplitude (pA)	3R	< .001	0.034	Kruskal-Wallis test	0.030	Dunn’s test	0.666	0.018	0.024

mEPSC rise time (ms)	3S	0.245		One-way ANOVA	0.055				
mEPSC decay time (ms)	3T	< .001	0.075	One-way ANOVA※	0.071				
mEPSC half width (ms)	3U	0.004	0.162	One-way ANOVA※	0.013	Tukey's HSD test	0.039	0.997	0.032

*NLT: Natural Logarithm Transformation of the data

One-way ANOVA※ ：One-way ANOVA after natural logarithm transformation of the data

Supplementary table 2. Comparison of electrophysiological properties between group-housing mice and Isolate-housing mice

Variable	Figure	Cell subclass	Shapiro-Wilk test	Shapiro-Wilk test after NLT*	Statistics	
					Statistical methods	p value
Sag ratio (%)	4B	MD			Kolmogorov-Smirnov test	0.395
Sag ratio (%)	4B	STR			Kolmogorov-Smirnov test	0.067
Sag ratio (%)	4B	PON			Kolmogorov-Smirnov test	0.832
Input resistance (Mohm)	4C	MD	0.313		Student's t test	0.120
Input resistance (Mohm)	4C	STR	0.091		Student's t test	0.927
Input resistance (Mohm)	4C	PON	< .001	< .001	Mann-Whitney's U test	0.208
Rheobase (pA)	4D	MD	< .001	0.052	Student's t test※	0.038
Rheobase (pA)	4D	STR	< .001	0.009	Mann-Whitney's U test	0.686
Rheobase (pA)	4D	PON	< .001	0.215	Student's t test※	0.208
Spike threshold (mV)	4E	MD	0.471		Student's t test	0.238
Spike threshold (mV)	4E	STR	0.543		Student's t test	0.908
Spike threshold (mV)	4E	PON	0.016	0.002	Mann-Whitney's U test	0.488
Spike amplitude (mV)	4F	MD	0.040	0.007	Mann-Whitney's U test	0.443
Spike amplitude (mV)	4F	STR	0.033	0.002	Mann-Whitney's U test	0.923
Spike amplitude (mV)	4F	PON	0.002	< .001	Mann-Whitney's U test	0.214
Spike upstroke (V/s)	4G	MD	0.547		Student's t test	0.125
Spike upstroke (V/s)	4G	STR	0.078		Student's t test	0.727
Spike upstroke (V/s)	4G	PON	0.904		Student's t test	0.214
Spike downstroke (V/s)	4H	MD	0.527		Student's t test	0.981
Spike downstroke (V/s)	4H	STR	0.632		Student's t test	0.078
Spike downstroke (V/s)	4H	PON	0.722		Student's t test	0.379
Spike frequency (Hz)	4I	MD	0.119		Student's t test	0.122
Spike frequency (Hz)	4I	STR	0.027	< .001	Mann-Whitney's U test	0.795
Spike frequency (Hz)	4I	PON	0.016	0.794	Student's t test※	0.812
Last/first ISI ratio	4J	MD	< .001	< .001	Mann-Whitney's U test	0.731
Last/first ISI ratio	4J	STR	< .001	0.001	Mann-Whitney's U test	0.205
Last/first ISI ratio	4J	PON	< .001	0.001	Mann-Whitney's U test	0.381
Spike frequency-current curves	4K	MD			MANOVA	0.334
Spike frequency-current curves	4L	STR			MANOVA	0.233
Spike frequency-current curves	4M	PON			MANOVA	0.252
Input resistance (Mohm)	4N	MD-PH	0.060		Student's t test	0.872
Input resistance (Mohm)	4N	STR-PH	0.068		Student's t test	0.456
Input resistance (Mohm)	4N	PON-PH	< .001	0.001	Mann-Whitney's U test	0.321
Rheobase (pA)	4O	MD-PH	0.028	0.512	Student's t test※	0.004
Rheobase (pA)	4O	STR-PH	0.120		Student's t test	0.334
Rheobase (pA)	4O	PON-PH	< .001	0.087	Student's t test※	0.888

Spike threshold (mV)	4P	MD-PH	0.903		Student's t test	0.052
Spike threshold (mV)	4P	STR-PH	0.010	0.001	Mann-Whitney's U test	0.051
Spike threshold (mV)	4P	PON-PH	0.006	< .001	Mann-Whitney's U test	0.589
Spike amplitude (mV)	4Q	MD-PH	0.075		Student's t test	0.554
Spike amplitude (mV)	4Q	STR-PH	0.124		Student's t test	0.416
Spike amplitude (mV)	4Q	PON-PH	< .001	< .001	Mann-Whitney's U test	0.330
Spike upstroke (V/s)	4R	MD-PH	0.511		Student's t test	0.663
Spike upstroke (V/s)	4R	STR-PH	0.737		Student's t test	0.385
Spike upstroke (V/s)	4R	PON-PH	0.698		Student's t test	0.574
Spike downstroke (V/s)	4S	MD-PH	0.680		Student's t test	0.451
Spike downstroke (V/s)	4S	STR-PH	0.996		Student's t test	0.182
Spike downstroke (V/s)	4S	PON-PH	0.612		Student's t test	0.414
Spike frequency (Hz)	4T	MD-PH	0.800		Student's t test	0.083
Spike frequency (Hz)	4T	STR-PH	0.020	< .001	Mann-Whitney's U test	0.073
Spike frequency (Hz)	4T	PON-PH	0.018	0.812	Student's t test※	0.695
Last/first ISI ratio	4U	MD-PH	0.164		Student's t test	0.266
Last/first ISI ratio	4U	STR-PH	0.023	0.027	Mann-Whitney's U test	0.458
Last/first ISI ratio	4U	PON-PH	< .001	0.003	Mann-Whitney's U test	0.345
Spike frequency-current curves	4V	MD-PH			MANOVA	0.039
Spike frequency-current curves	4W	STR-PH			MANOVA	0.468
Spike frequency-current curves	4X	PON-PH			MANOVA	0.444
sEPSC frequency (Hz)	5A	MD	< .001	0.377	Student's t test※	0.818
sEPSC frequency (Hz)	5A	STR	0.001	0.509	Student's t test※	0.886
sEPSC frequency (Hz)	5A	PON	0.015	0.371	Student's t test※	0.044
sEPSC amplitude (pA)	5B	MD	< .001	< .001	Mann-Whitney's U test	0.825
sEPSC amplitude (pA)	5B	STR	< .001	0.006	Mann-Whitney's U test	0.213
sEPSC amplitude (pA)	5B	PON	< .001	0.127	Student's t test※	0.045
mEPSC frequency (Hz)	5C	MD	< .001	0.333	Student's t test※	0.668
mEPSC frequency (Hz)	5C	STR	< .001	0.892	Student's t test※	0.782
mEPSC frequency (Hz)	5C	PON	0.026	0.609	Student's t test※	0.635
mEPSC amplitude (pA)	5D	MD	0.117		Student's t test	0.113
mEPSC amplitude (pA)	5D	STR	< .001	0.078	Student's t test※	0.206
mEPSC amplitude (pA)	5D	PON	< .001	0.017	Mann-Whitney's U test	0.803
sEPSC frequency (Hz)	5E	MD-PH	0.013	0.242	Student's t test※	0.499
sEPSC frequency (Hz)	5E	STR-PH	0.006	0.741	Student's t test※	0.922
sEPSC frequency (Hz)	5E	PON-PH	0.018	0.611	Student's t test※	0.036
sEPSC amplitude (pA)	5F	MD-PH	0.076		Student's t test	0.850
sEPSC amplitude (pA)	5F	STR-PH	0.010	0.227	Student's t test※	0.147
sEPSC amplitude (pA)	5F	PON-PH	< .001	0.073	Student's t test※	0.073
mEPSC frequency (Hz)	5G	MD-PH	0.010	0.423	Student's t test※	0.306
mEPSC frequency (Hz)	5G	STR-PH	< .001	0.332	Student's t test※	0.489
mEPSC frequency (Hz)	5G	PON-PH	0.068		Student's t test	0.340
mEPSC amplitude (pA)	5H	MD-PH	0.098		Student's t test	0.168
mEPSC amplitude (pA)	5H	STR-PH	0.146		Student's t test	0.127

mEPSC amplitude (pA)	5H	PON-PH	< .001	0.016	Mann-Whitney's U test	0.785

*NLT: Natural Logarithm Transformation of the data

Student's t test※ ：Student's t test after natural logarithm transformation of the data

Supplementary table 3. The comparison of sEPSC frequency and amplitude between PH cell and non-PH cell in MD and STR of group-housing mice

Cell type	Variable	Group	Mean	SD	Statistics
MD	sEPSC frequency (Hz)	PH	3.62	1.918	Student's t test, t_{25} = 3.474, p = 0.002
		non-PH	1.605	0.861	
	sEPSC amplitude (mV)	PH	16.243	4.455	Student's t test, t_{25} = 2.186, p = 0.038
		nonPH	13.312	1.936	
STR	sEPSC frequency (Hz)	PH	2.683	2.048	Student's t test, t_{21} = 0.491, p = 0.629
		non-PH	2.243	1.257	
	sEPSC amplitude (mV)	PH	14.436	5.346	Student's t test, t_{21} = 0.537, p = 0.597
		non-PH	13.198	2.713	
